# Supplementary material for: Investigating the influence of physiologically relevant hydrostatic pressure on CHO cell batch culture
Source: Sci Rep. 2021 Jan 8;11:162. doi: 10.1038/s41598-020-80576-8 (PMC7794228; doi:10.1038/s41598-020-80576-8)
Supplement: Supplementary file 1 — Supplementary Information 1. [file 41598_2020_80576_MOESM1_ESM.zip › S1/FLEX2/13Jan 30mmHg SS1 is C/Jan12 SS1.pdf]

===

```
Acq. Operator   : SYSTEM                         Seq. Line :   6
Sample Operator : SYSTEM
Acq. Instrument : HPLC-0039                       Location :   14
Injection Date  : 1/14/2019 4:12:23 PM                 Inj :   1
                                               Inj Volume : 100.000 µl
Sequence File   : C:\Users\Public\Documents\ChemStation\1\Data\TK_CHO_IgG 2019-01-14 15-49-10
                  \TK_CHO_IgG.S
Method          : C:\Users\Public\Documents\ChemStation\1\Data\TK_CHO_IgG 2019-01-14 15-49-10
                  \CHO_IgG_LC.M (Sequence Method)
Last changed    : 12/4/2018 1:38:41 PM by SYSTEM
```

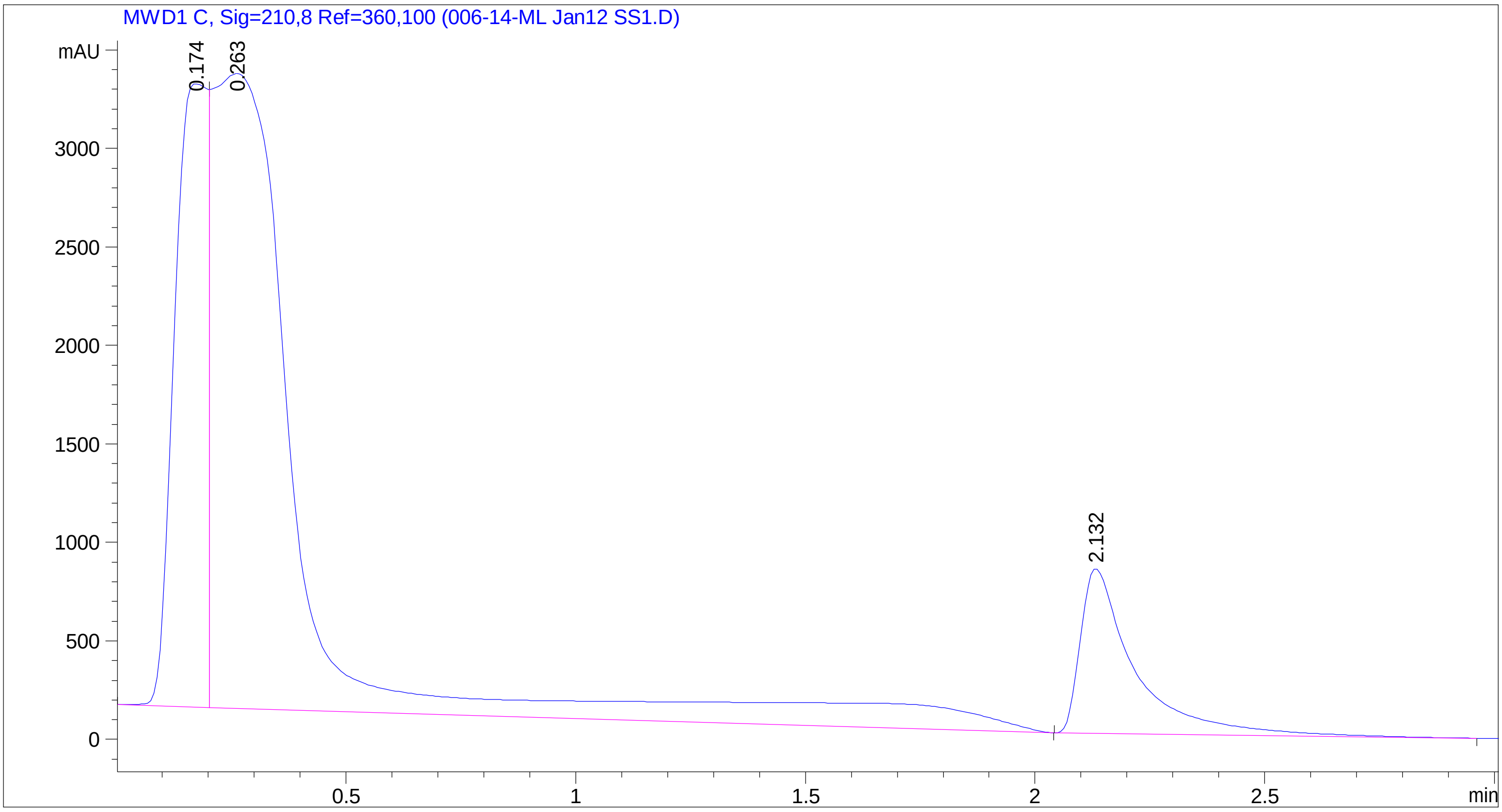

===

Area Percent Report

===

```
Sorted By             :      Signal
Multiplier            :      1.0000
Dilution              :      1.0000
Use Multiplier & Dilution Factor with ISTDs
```

Signal 1: MWD1 C, Sig=210,8 Ref=360,100

Peak #	RetTime [min]	Type	Width [min]	Area [mAU*s]	Height [mAU]	Area %
1	0.174	BV	0.0790	1.54593e4	3163.65698	23.8173
2	0.263	VB	0.1901	4.25642e4	3222.13623	65.5764
3	2.132	BBA	0.1161	6884.31836	835.58350	10.6063

Totals : 6.49079e4 7221.37671

===

*** End of Report ***
